# Changes in the identification and management of mental health and domestic abuse among pregnant women during the COVID-19 lockdown: regression discontinuity study

**DOI:** 10.1192/bjo.2022.66

**Published:** 2022-06-03

**Authors:** Rosanna Hildersley, Abigail Easter, Ioannis Bakolis, Lauren Carson, Louise M. Howard

**Affiliations:** Section of Women's Mental Health, Department of Health Service and Population Research, Institute of Psychiatry, Psychology and Neuroscience, King's College London, UK; Section of Women's Mental Health, Department of Health Service and Population Research, Institute of Psychiatry, Psychology and Neuroscience, King's College London, UK; and Department of Women and Children's Health, School of Life Course Sciences, King's College London, UK; Department of Biostatistics and Health Informatics, Institute of Psychiatry, Psychology and Neuroscience, King's College London, UK; and Centre for Implementation Science, Department of Health Service and Population Research, Institute of Psychiatry, Psychology and Neuroscience, King's College London, UK; Department of Women and Children's Health, School of Life Course Sciences, King's College London, UK; and Department of Psychological Medicine, Institute of Psychiatry, Psychology and Neuroscience, King's College London, UK; Department of Women and Children's Health, School of Life Course Sciences, King's College London, UK

**Keywords:** Perinatal psychiatry, epidemiology, depressive disorders, out-patient treatment, statistical methodology

## Abstract

**Background:**

Domestic violence and abuse (DVA) and mental illness during pregnancy have long-lasting and potentially serious consequences, which may have been exacerbated during the COVID-19 pandemic.

**Aims:**

To investigate how the UK COVID-19 lockdown policy influenced the identification of DVA and depressive symptoms during pregnancy in health services in South-East London in Spring 2020, using eLIXIR (Early-Life Data Cross-Linkage in Research) maternity and mental routine healthcare data.

**Method:**

We used a regression discontinuity approach, with a quasi-experimental study design, to analyse the effect of the transition into and out of the COVID-19 lockdown on the rates of positive depression screens, DVA recorded in maternity and secondary mental health services, and contact with secondary mental health services during pregnancy.

**Results:**

We analysed 26 447 pregnancies from 1 October 2018 to 29 August 2020. The rate of DVA recorded in maternity services was low throughout the period (<0.5%). Within secondary mental health services, rates of DVA dropped by 78% (adjusted odds ratio 0.219, *P* = 0.012) during lockdown, remaining low after lockdown. The rate of women screening positive for depression increased by 40% (adjusted odds ratio 1.40, *P* = 0.023), but returned to baseline after lockdown lifted.

**Conclusions:**

Rates of DVA identification in secondary mental health services dropped during and after lockdown, whereas overall rates of DVA identified in maternity services were concerningly low. Healthcare services must adopt guidance to facilitate safe enquiry, particularly in remote consultations. Further research is vital to address the longer-term impact on women's mental health caused by the increase in depression during the lockdown.

The steps taken to mitigate the spread of COVID-19 globally have had wide-reaching consequences for both physical and mental health. Changes to health and social care services, employment and social situations have raised concerns about the effects of these restrictions on mental health^1,2^ and domestic violence and abuse (DVA).^3,4^ In the UK, a nationwide ‘lockdown’ policy was ordered on 23 March 2020, requiring people to stay at home as much as possible, non-essential businesses were closed and people from different households were restricted from mixing.^5^ Pregnant women were advised to ‘shield’, meaning that they should stringently follow the lockdown guidelines and stay at home in all circumstances, unless seeking medical care.^6^ The consequential isolation and economic instability, along with fear relating to contracting COVID-19, made pregnant women especially at risk of mental health problems and DVA.^2,7,8^ Furthermore, the shift to telehealth for obstetric and mental healthcare may have impeded healthcare providers’ ability to identify women at risk.^9^ It is recommended that all women are asked about DVA during their antenatal care. However, there are several barriers to disclosing and identifying DVA, and the observed identification rate is likely to be lower than the estimated population prevalence (e.g. 4–8% during pregnancy in high-income countries).^10^ Furthermore, given the increased risk of DVA among women who experience mental illness, the rate of DVA identified within mental health services is likely to be substantially higher than other health and care services. A confidential enquiry into maternal deaths during the initial phase of restrictions in the UK highlighted the lack of identification and response to ‘red flags’. These red flags included rapidly escalating symptoms, previous history of severe mental illness and women's self-reported distress, which were missed because of changes in policy in response to COVID-19, shifting away from in-person assessments. These factors were shown to have potentially contributed to the deaths of six pregnant or postnatal women owing to suicide or DVA during March and May 2020.^11^ At the time of writing, there is limited evidence relating to any change in rate of pregnant women seeking help from services for mental disorders or DVA, and most evidence relating to changes in women's mood during the lockdown has been derived from surveys. These surveys are subject to recruitment biases that are not present in routinely collected data.

## Aims and objectives

We therefore aimed to explore the impact of the introduction and lifting of the UK COVID-19 lockdown policy on (a) the rate of DVA recorded during pregnancy in maternity and secondary mental healthcare services; (b) the rate of identified and recorded depressive symptoms in maternity services; and (c) the rate of referrals to, and type of contact with, secondary mental health services before the first UK lockdown on 23 March 2020, during the lockdown and following an easing of restrictions on 10 May 2020. In addition, we aimed to explore whether the impact of the UK COVID-19 lockdown policy on rates of depressive symptoms and DVA disproportionately affected women living in areas of high deprivation or from Black, Asian and minority ethnic backgrounds.^2,12,13^

## Method

### Data sources

This study utilises linked maternity and mental health records held within the Early Life Cross-Linkage in Research (eLIXIR) Partnership database. The eLIXIR Partnership is a unique repository of real-time pseudonymised data extracted from the electronic healthcare records of two acute and one mental health National Health Service (NHS) Trust in South London.^14^

Maternity and neonatal data for two South London NHS acute trusts are recorded on the BadgerNet electronic patient record system (CleverMed). This system contains data on community and hospital appointments during pregnancy and in the early postnatal period. The system captures data from clinical records, including maternity clinical data, demographic, and physical and mental health history. Mental health records were obtained from the South London and Maudsley NHS Foundation Trust (SLaM) Clinical Records Interactive Search (CRIS) system.^15^ The CRIS system generates variables extracted from electronic mental health records in SLaM extracted from both structured and open-text entries, using natural language processing (NLP) applications. The composition of the data-set and temporality of data collection is further described in Supplementary Figure 1 available at https://doi.org/10.1192/bjo.2022.66. Selected variables from the SLaM CRIS data-set and BadgerNet systems, linked at the individual level as part of the eLIXIR partnership database, were extracted. Maternity data were extracted for the period from 1 October 2018 to 29 August 2020, and mental health service data were available from 1 January 2008 to 14 December 2020, which represented the entire timespan available at the time of data extraction.

In this study, we define the pre-lockdown period as before 23 March 2020, the lockdown period as the time from 23 March 2020 to 10 May 2020, and the post-lockdown period as the time from 11 May 2020 to 29 August 2020. For outcomes measured at women's first antenatal appointment, women were grouped into these epochs based on their first antenatal appointment date. For outcomes measured within secondary mental health services, women were grouped on the basis of the date they were referred to mental health services. A further description of the composition of the data-set can be found in the Supplementary Appendix 1 and Supplementary Figure 1.

### Antenatal data variables

#### Descriptive sociodemographic characteristics

Maternity (i.e. weeks’ gestation at booking, parity, gravida, body mass index at booking and late booking status) and sociodemographic (i.e. maternal age, ethnicity, Index of Multiple Deprivation (IMD),^16^ employment status at booking, if women need interpreters and if women have no recourse to public funds) data were recorded by midwives and extracted from women's antenatal booking appointment records.

As part of routine assessment, women are asked at their antenatal booking appointment about current or past physical and mental health and DVA, alongside several questions related to socioeconomic status. This information was extracted from BadgerNet through the eLIXIR partnership database linkage.

#### Depression and DVA variables measured in maternity services

The Whooley questions (two depression screening questions) consist of a yes/no response and are used in routine practice.^17^ Women's responses were considered as Whooley positive if they answered yes to one or both questions. The Whooley questions have been found to have high specificity and moderate sensitivity in identifying antenatal depression, and are a valuable tool for identifying possible cases of a range of mental health conditions in early pregnancy.^18^ Also, women responding positively to one or both questions were asked if they required help with the difficulties identified. As the rate of missingness in this variable was low (3.38% of women were missing answers to one or both of the Whooley questions), we omitted observations with missing data for both questions from the analysis relating to this variable. Women who answered positively to one question and were missing data for the other question were counted as Whooley positive, and women who answered negatively to one question and were missing data for the other question were counted as Whooley negative.

Experiences of DVA were recorded by the midwife at the antenatal booking appointment or first contact with maternity services were extracted from BadgerNet. Experiences of DVA were identified by a text search of a semi-structured variable recording women's ‘sensitive risk factors’, which is derived from a semi-structured field within the antenatal notes. This variable contains several standard phrases recorded within the notes, with the case-sensitive phrase ‘domesticAbuse’ indicating that the midwife recorded that the participant had reported experiencing DVA at their first antenatal appointment.

### Mental health service use variables

Data on referral and contact with the local secondary mental health service in the 4 months after women's antenatal booking appointment were extracted from the CRIS system. These data were constructed as two different variables: one binary variable indicating if a women had any virtual (or face-to-face) contact with secondary mental health services during pregnancy, and one continuous variable indicating how many virtual (or face-to-face) contacts they had with secondary mental health services during pregnancy. The number of face-to-face contacts, virtual contacts, date of referral during pregnancy and total number of women in contact with these services during this time were extracted.

The binary indicator DVA recorded in secondary mental health records (yes/no) were extracted with NLP applications previously validated (interrater reliability 87%; using search terms ‘Domestic Violence’ and ‘DV’) for use in the CRIS data-set.^19^ No details relating to the character or severity of DVA are currently available in this data-set, and the definition used includes psychological, physical and sexual abuse of women from previous or current partners, as well as other family members.

### Statistical analyses

Descriptive statistics of sociodemographic, antenatal characteristics and mental health characteristics over the pre-lockdown, during lockdown and post-lockdown groups were estimated.

Regression discontinuity is a quasi-experimental design that exploits the introduction of a widespread change within a population (i.e. the intervention) at a clearly delineated threshold date, and creates comparable populations with different exposure to the intervention before and after the intervention. In this study, the introduction of the COVID-19 lockdown policy on 23 March 2020 acts as the first intervention of interest, followed by the start of lifting of the COVID-19 lockdown policy on 10 May 2020. The outcome(s) of interest must be continually measured both before and after the intervention(s), to allow for direct comparison between the groups who have or have not been exposed to the intervention (here, the pre-lockdown, during lockdown and post-lockdown groups), and the delineation between the group exposed to the intervention and the group not exposed to the intervention should act as a randomisation tool. The key feature of regression discontinuity design is the focus on comparing outcomes in a ‘short’ time interval before the intervention with a ‘short’ time interval after the intervention. By using these short time windows, we can assume that no unobserved factors confound the relationship between the exposure and the outcome in that short time interval.

In our analysis, we employed a fuzzy regression discontinuity analysis approach to account for some variation in the employment of the COVID-19 lockdown policies around the cut-off dates. In our analysis, we deployed a fuzzy regression discontinuity study design combined with a difference-in difference approach to estimation,^20^ comparing measures before and after the lockdown announcement in 2020 to those before and after the same date in 2019, allowing us to account for potential seasonal changes by effectively using the data from 2019 as a control group.^21^ A similar analysis was conducted for the lift of lockdown announcement. Further details regarding the assignment of observations to pre-lockdown, during lockdown and post-lockdown groups can be found in Supplementary Appendix 1: Data extraction and timepoints in pregnancies, and further details of the fuzzy regression discontinuity study design can be found in Supplementary Appendix 2: Technical appendix. We used logistic and ordinary least squares regression modelling to assess the changes associated with the introduction and lift of the lockdown policy intervention in binary and continuous outcome variables, respectively.

To test the variation in the impact of the UK lockdown on pregnant women in minority ethnic groups, or living in areas with increased levels of deprivation (i.e. low IMD scores), we conducted a further subgroup analysis. Using the fuzzy regression discontinuity analysis method, we tested the impact of the UK lockdown on the likelihood that women had a positive Whooley screen or were referred to secondary mental health services, by ethnicity and IMD quintile. Further subgroup analyses, e.g. to explore potential variation in rates of recorded DVA, were not conducted because of the sample size.

Sensitivity analyses were performed to test the modelling, and details of the tests used are outlined in Supplementary Appendix 3: Sensitivity analysis.

All analysis was conducted with Stata for Windows version 15 (StataCorp).

### Ethical approval

The authors assert that all procedures contributing to this work comply with the ethical standards of the relevant national and institutional committees on human experimentation and with the Helsinki Declaration of 1975, as revised in 2008. Ethics approval for the eLIXIR Partnership was granted by the Oxford C Research Ethics Committee (approval number 18/SC/0372). The Health Research Authority Confidential Advisory Group (approval number 18/CAG/0040) provided approval under Section 251 (s251) of the NHS Act (2006), for the eLIXIR partnership to provide a pseudonymised database for secondary analysis. This research project was approved by the eLIXIR oversight committee in November 2020 (reference: DL019).

This study was pre-registered with the COVID-19 and Mental Health Studies Register (https://www.maudsleybrc.nihr.ac.uk/research/covid-19-studies-project-details?id = 9673).

## Results

### Sample characteristics

**Fig. 1:**
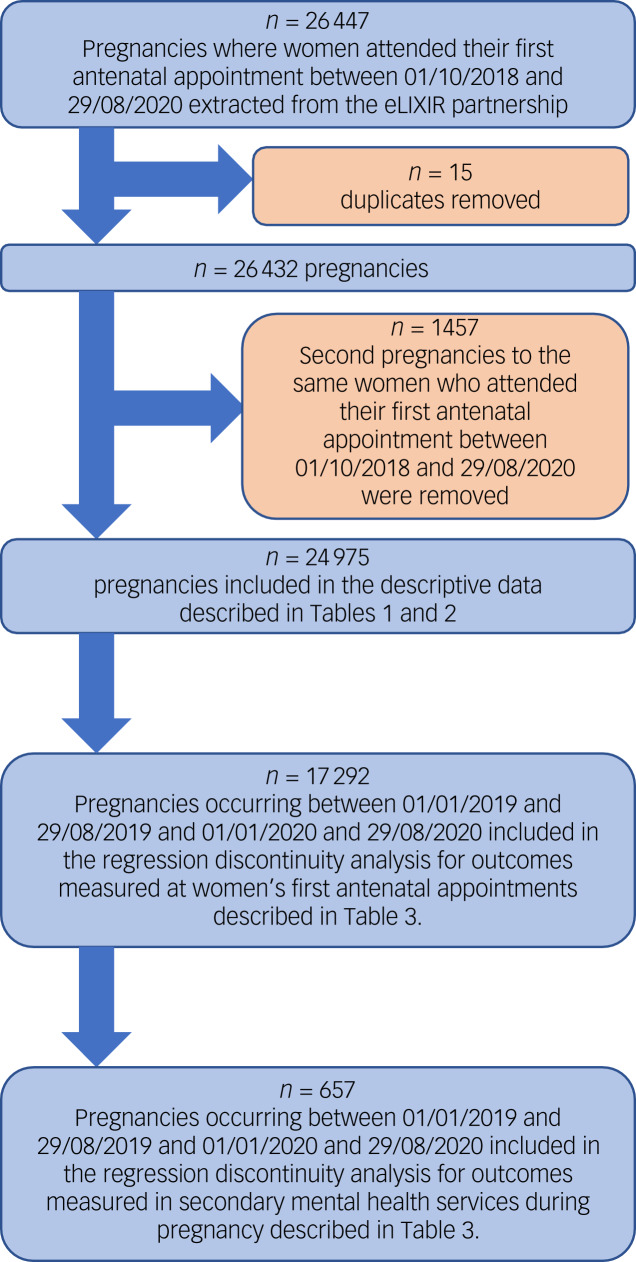
Flow chart showing excluded cases and which sections of the data-set were used in which analysis.

In total, data relating to 26447 pregnancies were extracted. First, completed duplicates were removed from the data-set (*n* = 15). A total of 1457 women had more than one pregnancy during the period from 1 October 2018 to 29 August 2020. The first pregnancy recorded within the eLIXIR database was considered the index pregnancy in this study. Data relating to later pregnancies were removed (*n* = 1457), to minimise bias relating to correlations between non-independent observations. Overall, 24 975 women with completed data from their antenatal booking appointment were included in the final data-set and analysis. Of these women, 991 were in contact with secondary care mental health services in South London during their pregnancy. In the full study cohort, 19 812 women had their first contact with maternity services between 1 October 2018 and 22 March 2020, and were included in the pre-lockdown group when analysing the descriptive data; 1684 had their first antenatal appointment between 23 March and 10 May 2020, and were included in the lockdown group; and 3479 had their first antenatal appointment between 11 May and 29 August 2020, after lockdown restrictions started to lift, and were included in the post-lockdown group. The regression discontinuity analysis included 17 292 women who attended their first antenatal appointment between 1 January and 29 August in both 2019 and 2020. The control group comprised 8169 women who attended their first antenatal appointment in 2020 and 9123 women who attended their first antenatal appointment in 2019. A flow chart describing which cases were included in which part of the analysis is shown in Fig. 1.

The characteristics of the sample are shown in Table 1, and the descriptive data relating to the outcomes measured are shown in Table 2.

**Table 1:** Descriptive statistics of sociodemographic and antenatal characteristics of the full study cohort

	Pre-lockdown (1 Oct 2018–22 Mar 2020)		Lockdown (23 Mar 2020–10 May 2020)		Post-lockdown (11 May 2020–29 Aug 2020		Total cohort	
Variable	*n*	%	*n*	%	*n*	%	*N*	%
Total observations	19812		1684		3479		24975	
Mother's ethnicity								
Any other	1140	5.8%	139	8.3%	241	6.9%	1520	6.1%
South Asian	1274	6.4%	121	7.2%	196	5.6%	1591	6.4%
Black	4014	20.3%	316	18.8%	613	17.6%	4943	19.8%
White	9745	49.2%	814	48.3%	1637	47.1%	12196	48.8%
Chinese	383	1.9%	41	2.4%	78	2.2%	502	2.0%
Mixed ethnicity	899	4.5%	78	4.6%	170	4.9%	1147	4.6%
Unknown	2357	11.9%	175	10.4%	544	15.6%	3076	12.3%
IMD quintile								
1 (most deprived)	3786	19.1%	316	18.8%	675	19.4%	4777	19.1%
2	8111	40.9%	716	42.5%	1476	42.4%	10303	41.3%
3	4964	25.1%	424	25.2%	854	24.5%	6242	25.0%
4	1874	9.5%	143	8.5%	311	8.9%	2328	9.3%
5 (least deprived)	832	4.2%	60	3.6%	130	3.7%	1022	4.1%
Employment status								
Other	1138	5.7%	118	7.0%	210	6.0%	1466	5.9%
Employed (including full time, part time and maternity leave)	13583	68.6%	1194	70.9%	2443	70.2%	17220	68.9%
Student	422	2.1%	36	2.1%	71	2.0%	529	2.1%
Full-time mother/carer	1306	6.6%	89	5.3%	187	5.4%	1582	6.3%
Unemployed	2356	11.9%	199	11.8%	480	13.8%	3035	12.2%
Not recorded	1016	5.1%	48	2.9%	86	2.5%	1150	4.6%
Interpreter required	1274	5.5%	114	6.8%	245	7.0%	1633	6.5%
No recourse to public funds	1096	5.5%	95	5.6%	219	6.3%	1410	5.6%
Booked at the weekend	4718	23.8%	490	29.1%	719	20.7%	5927	24%
Late booking (after 12 + 6 weeks)	4377	22.1%	263	19.6%	607	17.5%	5247	21%
	Mean	s.d.	Mean	s.d.	Mean	s.d.	Mean	s.d.
Maternal age at first antenatal appointment (years)	32.68	5.51	33.0	5.4	32.68	5.4	32.7	5.49
Maternal BMI^a^	25.45	5.4	25.23	6.41	24.97	5.37	25.39	5.45
Gestational age at booking (weeks)	11.6	6.53	10.31	5.48	10.33	5.48	11.35	6.55
	Median	IQR	Median	IQR	Median	IQR	Median	IQR
Parity	0	1	0	1	0	1	0	1
Gravida	2	2	2	2	2	2	2	2

IMD, Index of Multiple Deprivation; BMI, body mass index; IQR, interquartile range.

The IQR shown in the table is the difference between the 25th and 75th percentile (1st and 3rd quarter), in line with reporting guidence for IQRs.

a. Maternal BMI was very poorly measured at women's first antenatal appointments with 13% overall missing, 42.3% missing during lockdown and 38.4% missing post-lockdown.

**Table 2:** Descriptive statistics of mental health and domestic violence and abuse outcomes

	Pre-lockdown (1 Oct 2018–22 Mar 2020)		Lockdown (23 Mar 2020–10 May 2020)		Post-lockdown (11 May 2020–29 Aug 2020		Total cohort	
Variable	*n*	%	*n*	%	*n*	%	*N*	%
Total observations	19812		1684		3479		24975	
Mental health data collected at booking (BadgerNet)								
Whooley positive	1806	9.1%	179	10.6%	302	8.7%	2287	9.2%
Help required (recomended referral to Perinatal Mental Health Team, only asked if Whooley positive)	508	2.6%	36	2.1%	98	2.8%	642	2.6%
DVA recorded at booking	98	0.5%	<10	0.0%	16	0.5%	121	0.5%
Data collected in secondary mental health services (CRIS)								
Referral to secondary mental health services before pregnancy (percentage of total cohort)	810	4.1%	64	3.8%	117	3.4%	991	4.0%
DVA recorded during pregnancy by secondary mental health services^a^	264	32.6%	17	26.6%	43	36.8%	324	33%
Virtual contact made with secondary mental health services during pregnancy^a^	363	44.8%	42	65.6%	95	81.2%	500	50%
Face-to-face contact made with secondary mental health services during pregnancy^a^	564	69.6%	27	42.2%	49	41.9%	640	65%
	Median	IQR	Median	IQR	Median	IQR	Median	IQR
Frequency of face-to-face contact with secondary mental health services during pregnancy	2	5	2	2	2	2	2	5
Frequency of virtual contact with secondary mental health services during pregnancy	2	3	4	10	5	7	2	4

PMHT, Perinatal Mental Health Team; DVA, domestic violence and abuse; CRIS, Clinical Records Interactive Search; IQR, interquartile range.

a. Percentage of women referred to secondary mental health services.

#### Transition from pre-lockdown period to lockdown period

**Fig. 2:**
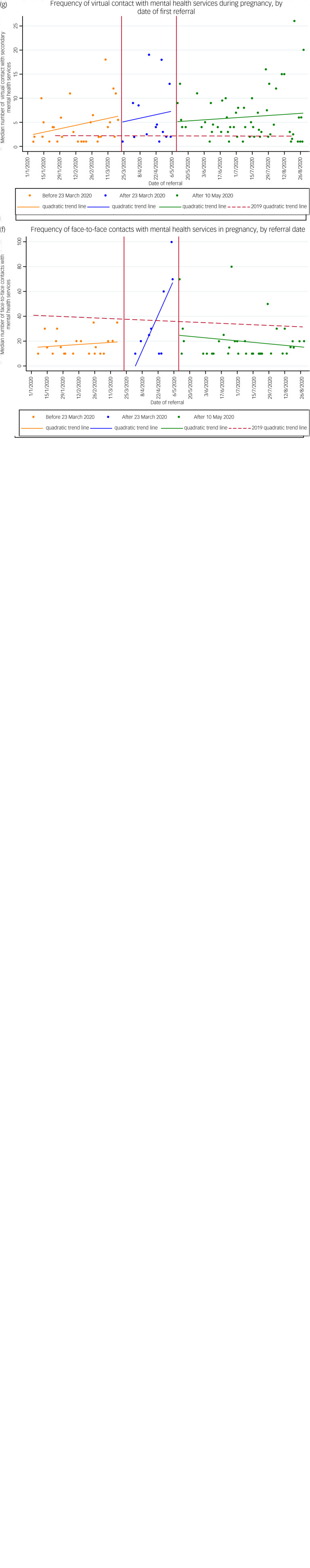
Data for before and after UK lockdown announcement on the following: (a) daily number of women identified as Whooley positive by booking date; (b) referral to secondary mental health services by booking date; (c) weekly rate of domestic violence and abuse (DVA) during the pregnancy period, recorded by secondary mental health services by referral date; percentage of women referred to secondary mental health services that had (d) face-to-face contact with secondary mental health services and (e) virtual contact with secondary mental health services, by week of referral; (f) daily median frequency of face-to-face contact with secondary mental health services, by the date of first face-to-face contact and (g) daily median frequency of virtual contacts with secondary mental health services, by the date of first virtual contact with secondary mental health services.

The results from this analysis provided evidence that there was an increase in the proportion of women who were Whooley positive between the pre-lockdown and during lockdown groups (adjusted odds ratio 1.40, 95% CI 1.05–1.87, *P* = 0.023; see Table 3). The rate of DVA recorded at women's first antenatal appointment was 0.5%, and the numbers reported during lockdown were similar, with all rates being too sparse to assess changes before and after lockdown. No other variables measured at women's first antenatal appointments showed a significant change, and there was no change in the odds that people were referred to secondary mental health services during pregnancy. Fig. 2 displays the data for before and after UK lockdown announcement for each of the outcomes, by the date of first virtual contact with secondary mental health services.

**Table 3:** Estimated effects of the start of lockdown on 23 March 2020 and lift of lockdown announcements on 10 May 2020 for women who attended their first antenatal appointment between 1 January and 29 August 2020, estimating transitions related to the lockdown announcement and lift-of-lockdown announcement

	Pre-lockdown versus lockdown	Pre-lockdown versus post-lockdown	Lockdown versus post-lockdown
	Adjusted odds ratio (95% CI)	Adjusted odds ratio (95% CI)	Adjusted odds ratio (95% CI)
Outcomes measured at first antenatal appointment			
Whooley positive^a^ *n* = 17 292	1.40 (1.05−1.87)*	0.92 (0.73−1.17)	0.66 (0.50−0.88)**
Help required^a^ *n* = 17 292	0.61 (0.307−1.21)	1.01 (0.593−1.73)	1.99 (0.86−3.24)
Antenatal DVA reported at booking^a^ *n* = 17 292	1.36 (0.38−4.92)	1.49 (0.52−4.30)	1.09 (0.33−3.62)
Referral to secondary mental health services during pregnancy^a^ *n* = 17 292	1.20 (0.77−1.86)	0.78 (0.55−1.12)	0.66 (0.43−1.00)
Outcomes measured in secondary mental health services			
Virtual contact with secondary mental health services during pregnancy (binary)^b^, *n* = 657	1.71 (0.57−5.19)	2.76 (1.25−6.07)*	1.61 (0.60−4.36)
Face-to-face contact with secondary mental health services during pregnancy (binary)^b^, *n* = 657	0.81 (0.26−2.52)	1.09 (0.49−2.42)	1.34 (0.39−3.66)
DVA recorded in secondary mental health services during pregnancy^b^, *n* = 657	0.22 (0.07−0.71)*	0.42 (0.18−0.98)*	1.92 (0.67−5.46)
	Correlation coefficient (95% CI)	Correlation coefficient (95% CI)	Correlation coefficient (95% CI)
Frequency of face-to-face contact with secondary mental health services during pregnancy^c^, *n* = 300	−0.02 (−0.41 to 4.02)	−0.67 (−3.43 to 2.09)	−0.66 (−4.35 to 3.04)
Frequency of virtual contact with secondary mental health services during pregnancy^c^, *n* = 329	0.36 (−0.37 to 4.41)	1.58 (−1.18 to 4.33)	1.22 (−2.16 to 4.89)

Adjusted odds ratios, correlation coefficients and 95% confidence intervals were estimated. DVA, domestic violence and abuse.

a. Results from logistic regression models adjusted for maternal ethnicity, monthly trends and trends over different days of the week, with the three cohorts calculated from the date of the first antenatal appointment.

b. Results from logistic regression models adjusted for monthly trends and trends over different days of the week, with the three cohorts calculated from the date of the first referral during pregnancy to secondary mental health services.

c. Results from linear regression models adjusted for monthly trends and trends over different days of the week with the three cohorts calculated from the date of the first referral during pregnancy to secondary mental health services.

*P < 0.05, **P < 0.01.

Women of all ethnicities and IMD quintiles with a large enough representation in our sample to allow for assessment showed an increased rate in the number of women who were Whooley positive. The increased rate of women who were Whooley positive during the lockdown period was largest among women who were White (compared with all other ethnic groups measured) (adjusted odds ratio 1.75, 95% CI 1.12–2.71, *P* = 0.013; see Supplementary Table 1.2) or in IMD quintile 4 (compared with all other IMD quintiles) (adjusted odds ratio 7.82, 95% CI 2.28–26.79, *P* = 0.001), where IMD quintile 1 represents the most deprived group and quintile 5 represents the least deprived group. It is also notable that there were nonsignificant increases in the odds that women of Black (adjusted odds ratio 1.72, 95% CI 0.95–3.14; see Supplementary Table 1.2) ethnicity were Whooley positive. However, the lack of statistical significance may be because of the smaller number of cases in this analysis rather than the absence of a change.

Among women in contact with secondary mental health services, there was a 78% decrease in the odds that DVA was recorded in mental healthcare records (adjusted odds ratio 0.22, 95% CI 0.07–0.71, *P* = 0.012) during lockdown, supporting our hypothesis that the rate of DVA identified in pregnancy would decrease. There was no evidence for change in the odds that contact with secondary mental health services during pregnancy was face to face or virtual, or in the frequency of contacts with mental health services during lockdown.

### Transition from lockdown period to post-lockdown period

There was a decrease in the number of Whooley-positive cases in the post-lockdown group compared with the lockdown group (adjusted odds ratio 0.66, 95% CI 0.50–0.88, *P* = 0.004; see Table 3), and the odds post-lockdown were comparable to the pre-lockdown odds (adjusted odds ratio 0.92, 95% CI 0.73–1.17, *P* = 0.518). No other variables measured at women's first antenatal appointments, including the rate of DVA recorded at women's first antenatal appointments, showed a sizeable change, and there was no change in the odds that women were referred to secondary mental health services during pregnancy when the post-lockdown group was compared with the pre-lockdown and lockdown groups, and this remained true for women of all ethnicities and IMD quintiles.

In the post-lockdown group, there was a decrease in the rate of DVA recorded in secondary mental health services during pregnancy (adjusted odds ratio 0.42, 95% CI 0.18–0.98, *P* = 0.043) compared with the pre-lockdown group, but no change when the lockdown cohort was compared with the post-lockdown cohort (adjusted odds ratio 1.92, 95% CI 0.67–5.46, *P* = 0.224), suggesting a sustained impact on the identification of DVA within secondary mental health services.

There was a significant increase in the virtual contact of women with secondary mental health services in the post-lockdown group compared with the pre-lockdown cohort (adjusted odds ratio 2.76, 95% CI 1.25−6.07, *P* = 0.012), but not when the post-lockdown group was compared with the lockdown group. There was no change in the odds that pregnant women had face-to-face contact with secondary mental health services. We detected no evidence of a change in the frequency of virtual or face-to-face contact with secondary mental health services. However, the descriptive statistics and graphical representations of the transition from lockdown indicate that there was an increase in the frequency of virtual contact with secondary mental health services in the post-lockdown group when compared with the pre-lockdown group; that is, women who were referred after lockdown had more virtual contacts with secondary mental health services than women who were referred before lockdown.

### Sensitivity analysis

The sensitivity analysis, described in full in Supplementary Appendix 3: Sensitivity analysis, and Supplementary Tables 2.1 and 2.2, highlighted the sensitivity to the cut-off dates used to assign observations to the pre-lockdown and during lockdown groups. Although the reduced reporting of DVA in secondary mental health services during pregnancy remained low, altering the cut-off dates reduced the odds ratio, indicating the rate at which women were Whooley positive when comparing the pre-lockdown and during lockdown groups.

## Discussion

### Key findings

Among women seen by mental health services, the odds that DVA was recorded during pregnancy decreased by approximately 78%. The rate of recorded DVA after lifting of lockdown appeared to increase slightly, but it did not return to pre-lockdown levels. There was evidence of an increase in the frequency of contacts using remote methods with secondary mental health services between the pre-lockdown period and during and after lockdown, which may explain the drop in recorded DVA because women may not have been able to safely disclose DVA when at home with the perpetrator. We identified no changes in rates of recorded DVA in maternity records of women who attended their first antenatal appointment in the periods before, during or after the UK lockdown, but the numbers of DVA recorded in maternity records were very low across all three time periods.

We also identified a 40% increase in the odds of women in contact for their first maternity appointment being identified as ‘Whooley’ (depression screen) positive during the first UK lockdown, which returned to the pre-lockdown rate after the lift of the lockdown on 11 May 2020. Although this did not persist following the easing of the lockdown measures, we do not know the long-term effects on women's mental health. The clear increase in rates of women who were Whooley positive during lockdown mirrors the observed and anticipated increase in new mental health issues and the deterioration of pre-existing mental ill health throughout the British population during the initial UK lockdown in the COVID-19 pandemic.^1,22,23^

However, there was no increase in the rate of referral to secondary mental health services during pregnancy, suggesting that maternity staff did not identify more cases of mental illness requiring secondary care or that they were more cautious of referring women.

### DVA

Emerging evidence suggests that DVA has increased during the COVID-19 pandemic in the UK and worldwide when considering crime statistics,^24–27^ helpline use^4^ or Google search trends,^28^ although a minority of studies have found little change in the rate of DVA or a reduction in cases reported to the police.^29–32^ Nonetheless, our findings relating to DVA recorded at booking and secondary mental health services during pregnancy are concerning. First, the rate of DVA recorded at women's first antenatal appointment falls well below the UK estimated prevalence of DVA experienced by pregnant women (approximately 2.5–9%)^33–36^ both before and during the pandemic. Second, it seems unlikely that the actual prevalence of DVA in the UK has decreased, so the reduction in recorded DVA during pregnancy in secondary mental health services is more likely to represent a lack of enquiry about DVA in women among healthcare professionals (possibly because of the known risk of asking remotely, with a perpetrator potentially present, and increasing risk of harm) or lack of disclosure during the pandemic. Further investigation would be needed to establish if the reduction in identification of DVA by healthcare providers was seen in other situations, such as general practitioner services. Similarly, DVA may be more readily identified in different services, such as emergency helplines, during the COVID-19 lockdown, which would not have been identified in our analysis. Nonetheless, at its worst, DVA during pregnancy is life-threatening: there were two maternal homicides in England during the UK lockdown period, with COVID-19-related disruption to services identifying and responding to DVA potentially preventing the protection of these women.^11^ Providers should be aware of advice from specialist organisations (e.g. https://irisi.org/wp-content/uploads/2020/06/Domestic-abuse-guidance-for-virtual-health-settings-C19.pdf) to adapt their practice around asking about DVA in virtual consultations, and ensuring women are asked at some point in pregnancy, when it is safe to do so, ideally more than once.^37^

### Mental health during the pregnancy period

Although significant, women who are Whooley positive in early pregnancy would require further assessment to establish whether they have a mental health disorder. The prevalence of women who were Whooley positive is notably lower than the rates of mental illness reported in surveys using similar methods to identify mental disorders during pregnancy during the pandemic, although current estimates of prevalence vary widely.^38–40^ One study estimated that, pre-pandemic, 15% of women screened positive for depression, whereas 40.7% were positive during the pandemic;^38^ other studies estimated that depression in pregnancy was present in approximately 12%^40^ or 15%^39^ of pregnant women during the pandemic.

At the population level, research during the pandemic has found that women aged between 18 and 34 years and people living with young children tended to have the greatest increase in mental health difficulties,^22^ and increases in adverse mental health effects were associated with insecure employment and low income.^23,41,42^ These issues are known risk factors for mental health disorders during pregnancy, and are likely to have worsened during the pandemic.^43^ However, we observed that the greatest increase in Whooley-positive cases was in women in IMD quintile 4, or the second least-deprived quintile, although it should be noted that our analysis of IMD quintiles 4 and 5 had limited power because of the sample size. Pan et al^44^ observed that people without prior mental health disorders showed the greatest increase in symptoms of mental disorders, which may be the reason for our observed increase in populations that previously had a lower incidence of mental health disorders during pregnancy. Nonetheless, it may be expected that the mental health of women with higher levels of deprivation may be more affected by the lockdown measures, as noted in other general population studies.^23,41,42^ Indeed, in our ethnically diverse population, most minority ethnic groups reported an increase in the proportion of women screening Whooley positive during the lockdown period.

At the time of writing, analysis of the duration of the effect of the COVID-19 pandemic and resultant mitigation strategies is ongoing, and evidence is sparse. However, some evidence is emerging that the population rates of anxiety and depression may have returned to baseline levels since the lift of lockdown measures.^45,46^ These findings are supported by our observation in the rebound of the rate of women screening Whooley positive after 10 May 2020, toward the pre-pandemic levels. However, it should be noted that our observations reflect the rate of recorded mental illness rather than the actual rate. Women may be cautious of reporting their struggle or asking for further help because of reduced services or contracting COVID-19 when accessing mental health services.^9^ This hesitancy may also be the reason underlying the lack of corresponding change in the responses to the ‘help required’ question at booking, or rate of referral to secondary mental health services. Overall, further research to establish the long lasting effects of the COVID-19 lockdown on population-level perinatal mental health will be essential, as the circumstances surrounding the COVID-19 pandemic are rapidly evolving, and it is probable that the effects on women's mental health will be similarly changeable.

### Changes in healthcare provision in secondary mental health services

It is unclear whether the decrease in the rate of DVA recorded within secondary mental health services is a direct result of disruption to service delivery or a more general effect of the pandemic on enquiry and disclosure of DVA within healthcare services. It is encouraging to see that face-to-face consultations were still provided for some women. Although there are some advantages to providing mental healthcare during pregnancy virtually, the drop in recorded DVA observed in this study may indicate that prolonged reliance on the virtual provision of care should be viewed cautiously.^9^ Both providers and patients have noted that virtual care can impede the patient–provider relationship and increase healthcare provision inequity in areas with poor internet access and digital poverty.^9,47–49^ In the future, perinatal mental health services should be cautious of relying on virtual consultations, and rather consider hybrid models of care tailored to the needs and situations of women.

### Strengths and limitations

An evident strength of our study is its diverse cohort drawn from healthcare registry data. Although the women included are from a single urban area, the inclusion of a wide range of ethnic and socioeconomic backgrounds represented in this study allows for broad generalisation. Furthermore, selection and recall bias inherent in many surveys is absent from this cohort. However, this study will more accurately reflect what women report to their healthcare providers rather than the actual incidence of events. Similarly, different parts of the UK experienced different durations of lockdown. For example, areas such as Greater Manchester and Leicester experienced a later lifting of the lockdown, and accordingly, application of the findings of this paper should be considered with this in mind.

The regression discontinuity analysis method allowed us to account for seasonal variation and effectively use the cohort from 2019 as a comparator. The nationwide UK lockdown provided a natural experiment to utilise this type of study design. However, because of limitations in our data and the ongoing nature of pregnancy, pinpointing the occurrence of variables within the cohort was challenging. For example, a woman may have had their booking appointment in January, before the COVID-19 pandemic became a widely recognised issue, but most of their pregnancy would have occurred during the UK lockdown. If the patient was not referred to secondary mental health services, we would have no data relating to how the lockdown affected their mood or experience of pregnancy. Even so, this study contributes an essential perspective of the effect of the COVID-19 pandemic on mental health and domestic violence during pregnancy.

Despite the large sample size, some of our analyses were hindered by a lack of power, particularly when looking at low rates such as DVA recorded within maternity settings. Further research is needed to determine the accuracy of the measure of DVA recorded at women's first antenatal appointment used in this paper. Also, referral to secondary mental healthcare during pregnancy was difficult to ascertain because of the limited window included in the lockdown period; therefore, the number of women captured by the data extraction process in this study for the lockdown and post-lockdown was limited. Furthermore, we do not have data relating to women referred to other secondary mental health providers, but this should not affect the overall change in rate of referral associated with the UK lockdown reported in this study.

Although the Whooley questions are a case finding rather than a diagnostic tool, the positive predictive value tends to be moderately high. As sensitivity for specific disorders is low,^18^ our estimate should not be taken as an indicator of true prevalence.

In conclusion, our findings suggest that there has been an increase in the rate of pregnant women suffering from some mental distress, but there is little evidence to support any increase in the rate of more severe mental illness requiring referral to secondary mental health services. The evidence suggesting that rates of women who were Whooley positive returned to baseline quickly is encouraging. Future studies would be needed to explore if these observations reflect an unmet need, or if the mental health consequences of the UK lockdown for pregnant women are less severe than previously feared.

Identification and documentation of DVA in pregnant women with severe mental illness has dropped during the pandemic, which is concerning considering the evidence suggesting that rates of DVA have increased. Future work is required to determine the reasoning for this, and in the meantime, healthcare providers should be careful to ask about DVA during consultations, when safe to do so.

Further work is planned to determine the medium-term effects of the UK lockdown on women's mental health after giving birth, and any relationships between perinatal mental health, the COVID-19 lockdown and birth outcomes.

## Data Availability

The data accessed by eLIXIR remain within an NHS firewall and governance is provided by the eLIXIR Oversight Committee reporting to relevant information governance clinical leads. Subject to these conditions, data access is encouraged, and those interested should contact the eLIXIR Chief Investigator (Professor Lucilla Poston; Lucilla.poston@kcl.ac.uk).

## Supplementary material

For supplementary material accompanying this paper visit https://doi.org/10.1192/bjo.2022.66. click here to view supplementary material
