# Supplementary material for: Changes in the identification and management of mental health and domestic abuse among pregnant women during the COVID-19 lockdown: regression discontinuity study
Source: BJPsych Open. 2022 Jun 3;8(4):e96. doi: 10.1192/bjo.2022.66 (PMC9171064; doi:10.1192/bjo.2022.66)
Supplement: Supplementary file 1 [file S2056472422000667sup001.docx]

Supplementary Materials

**Supplementary Materials 1: Data extraction and timepoints in pregnancies**

A timeline of how the data were extracted is displayed in Figure 1, which also expresses the challenge of establishing temporality within the data. For some pregnancies, this is straightforward. For example, pregnancy A, in Figure 1, the pregnancy and birth takes place in before lockdown and, although she has a postnatal referral to secondary mental health services, this paper is not discussing postnatal outcomes and is therefore classed as a routine pregnancy with no input from mental health services during pregnancy. However, pregnancy B in Figure 1, is more complicated. She was booked before the UK lockdown started, so her antenatal booking data was included within the pre-lockdown cohort. However, she was referred to secondary mental health services after the UK lockdown started, so her mental health data was included within the lockdown cohort. The birth data is currently unavailable, as she gave birth after 29^th^ August 2020, but her mental health data after this date, including, for example, the number of virtual contacts made during the pregnancy period, is available at the point of data extraction. To account for the way the timeline of pregnancy exists and the sudden nature of the COVID-19 pandemic in March 2020, we have attempted to select the most theoretically sound time points available within our dataset for the statistical analysis of the pre, during and post-lockdown effect on perinatal mental health. For example, in pregnancy B, data relating to her antenatal appointment, such as DVA reported at this appointment would be included in the pre-lockdown cohort for analysis, as her first antenatal appointment was on 03/03/2020. However, data relating to her secondary mental health care use, such as DVA recorded in secondary mental health services would be included in the post-lockdown cohort as she was referred on 01/10/2020.

Supplementary Materials Figure 1
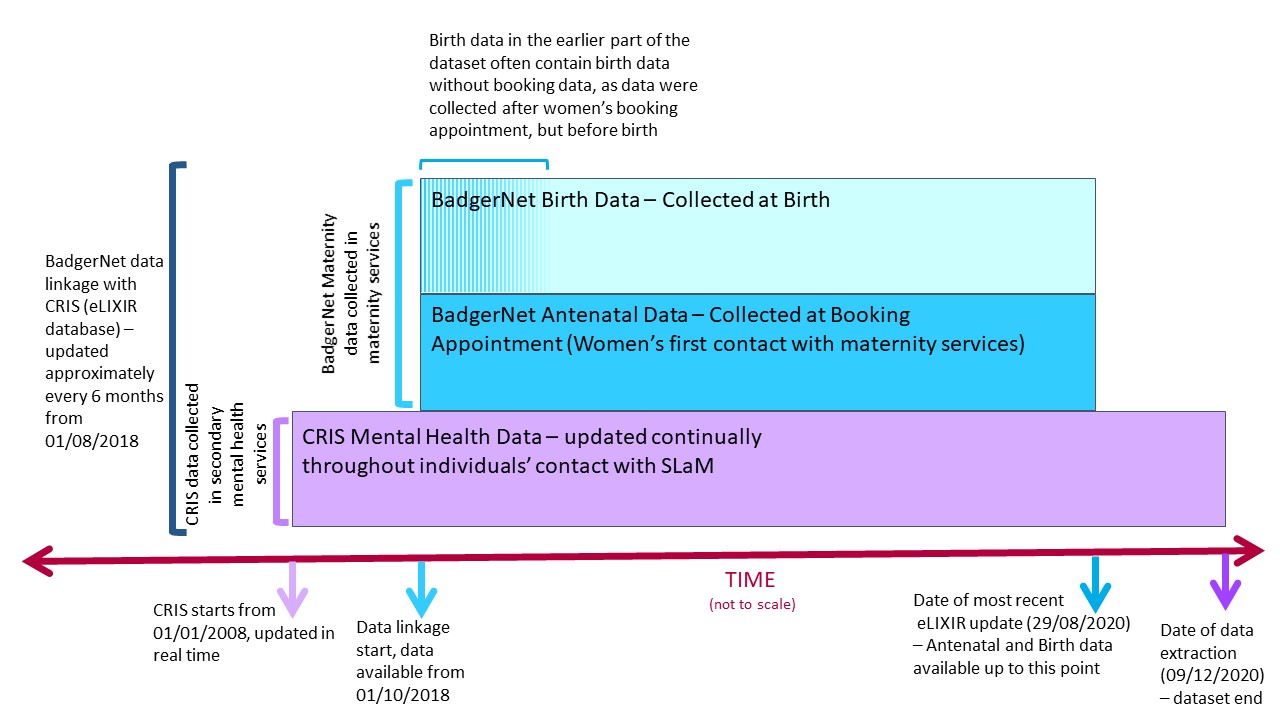

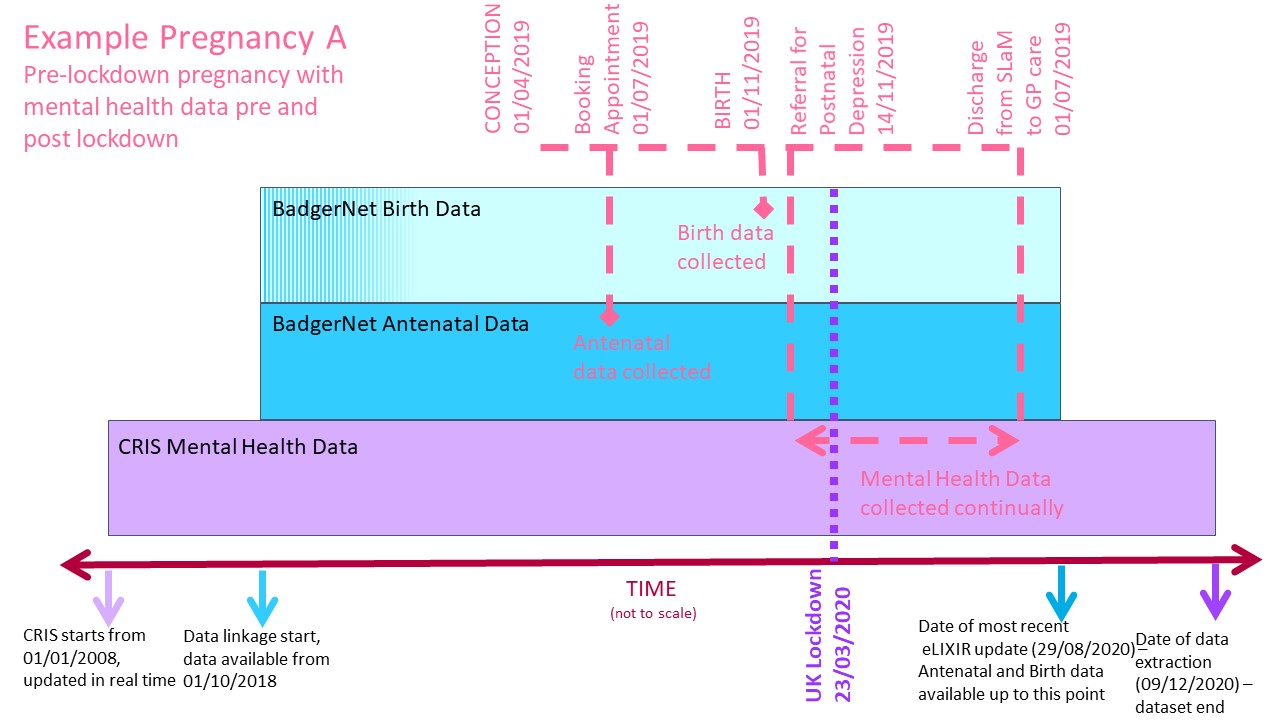


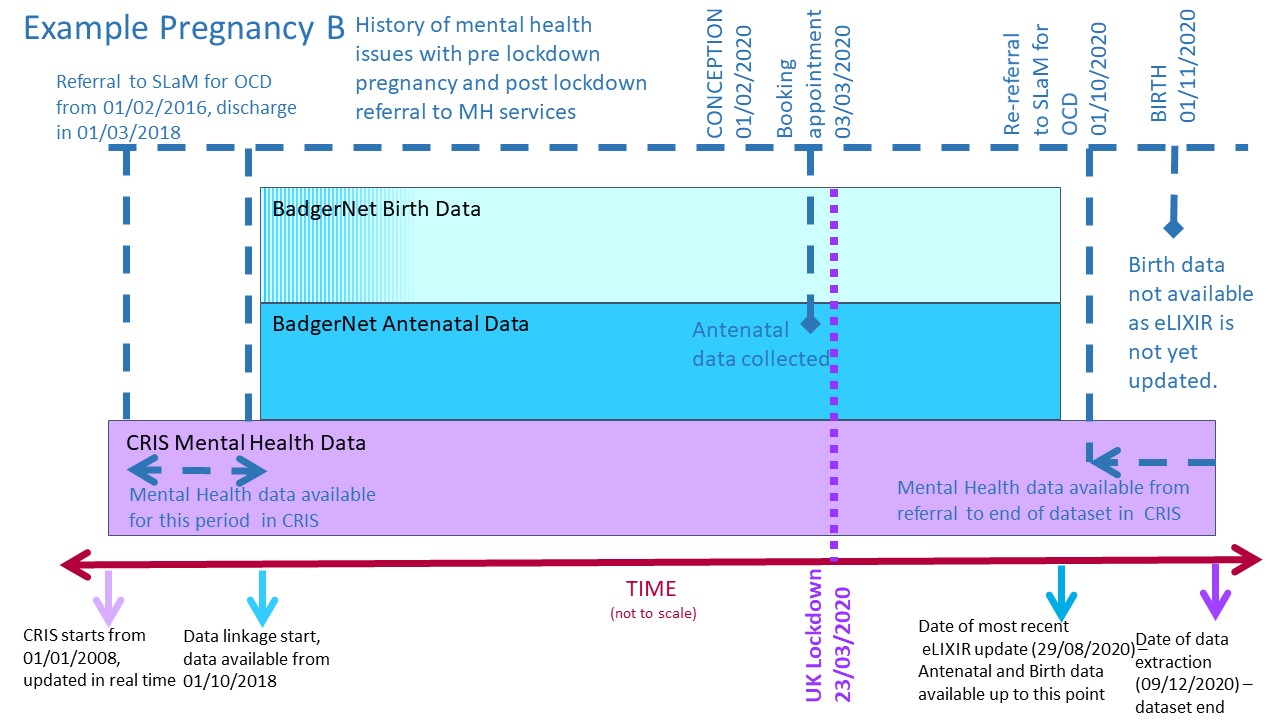


**Supplementary Materials 2: Technical Appendix - Further details and rationale for the statistical analysis**

The conditions for a valid FRD were met in our study^[[1]](#footnote-1)^: i) the decision rule (exposed or not exposed to the intervention) and cut-off value (23rd March2020 and10th May 2020) were known; ii) the assignment variable (distance from the lockdown announcement, measured in days) was continuous near the cut-off value and was not affected by the policy (see Figure 2 a-d in the paper); iii) there was visual confirmation of an intervention effect, in that a graphical analysis (Figure 2 a-d in the paper) confirmed the discontinuity, i.e. a visible jump at the cut-off value, indicating a policy effect. We also assumed parallel trends in the time window around the lockdown and lift of lockdown announcement (a form of common/parallel-trend assumption.

There are two types of RD design; a sharp regression discontinuity (SRD) design, applied when the probability of intervention assignment changes discontinuously at the cut-off date deterministically (from 0 to 1), and a fuzzy regression discontinuity (FRD) design, when the probability of intervention changes around the cut–off date stochastically. Due to the abrupt and clear changes in policy relating to COVID-19 and social distancing, all women would have effectively received the intervention (i.e. the mandatory UK lockdown).^5,6^ However, some may have already started working from home and taking social distancing procedures in the weeks before the start of the UK lockdown. To account for this uncertainty, we used a FRD design.

Differences in measures before versus after the cut-off dates were reported as odds ratios (ORs) and their corresponding 95% confidence intervals (CI). Logistic regression models were used to assess the effect of 23^rd^ March COVID-19 ' lockdown' policy on DVA and mental health service activity adjusted for temporal trends (e.g., weekday, month, year), sociodemographic variation between the three different cohorts (i.e., ethnicity), and taking into account overdispersion.

The variables analysed as part of the Fuzzy RD design data fulfilled parametric specifications for the conditional expectations. We also accounted for the heterogeneity of temporal effects (by weekday and month) to account for variation in reporting of the outcomes of interest (i.e., mental distress, measured as women who were Whooley positive at their first antenatal appointment; rate of Domestic Violence and Abuse (DVA) recorded in secondary mental health services etcetera). Within each pregnancy *i* we used logistic regression (for binary outcomes) or linear regression (for continuous outcomes) to model the odds of the woman experiencing one of the outcome variables (i.e., being Whooley positive, referral to secondary mental health services etcetera), defined as $Y_{i}$. $T_{i}$ is a categorical variable that describes if the woman attended her first antenatal appointment (or was referred to secondary mental health services or had contact with secondary mental health services) before lockdown (0), during lockdown (1) or after the lockdown (2), or during the equivalent periods in 2019. The year that the woman attended her antenatal appointment was a binary variable ${Year}_{i}$, corresponding to 2020. The model included the functions $E_{i}$ $W_{i}$ and $M_{i}$ as dummy variables to denote maternal ethnicity, weekday and month respectively.

$$Y_{i}= \beta_{0} {{+ \beta}_{1}T}_{i}{+ \beta}_{2}{Year}_{i}{{+ \beta}_{3}T}_{i}*{Year}_{i}{{+ \beta}_{4}W}_{i}{{+ \beta}_{5}M}_{i}{+ \beta}_{6}E_{i}+ \varepsilon_{i}$$

${\beta_{3}T}_{i}*{Year}_{i}$, or the interaction between the lockdown status (before, during or after lockdown) dummy variable $T_{i}$ and the year (2020 or 2019) variable ${Year}_{i}$ is the parameter of interest. This describes the effect of the lockdown on the odds or degree that that the outcome $Y_{i}$ is changed due to the intervention. The observations from 2019 effectively act as a control group. $\beta_{1}, \beta_{2},\ldots, \beta_{6}$ are fixed effects coefficients. Differences in the odds that an outcome occurred in a pregnancy or linear change in the outcome of interest before versus during or after the UK lockdown are reported as odds ratios (OR) or correlation coefficients for logistic and linear regression respectively.

**Supplementary Materials 3: Sensitivity analysis**

*Method*

A series of sensitivity analyses were performed to test the robustness of the regression modelling and sensitivity to the effects observed to the exact cut-off dates used to assign observations to pre-, during and post-lockdown cohorts for analysis.

- Altering the start date of the lockdown to 16th March 2020, when social distancing was advised.
- To account for anticipatory effects of the lockdown and lift-of-lockdown announcements, we omitted 1 week either side of the cut-off date of 23 March 2020 (ie., 16 March 2020 to 30 March 2020) and 1 week either side of 10 May 2020 (ie, 3 May 2020 to 17 May 2020). Anticipatory effects, such as the announcement of social distancing on 16 March 2020, could have resulted in altered MH service and DVA reporting activity in the study population just before the implementation of lockdown. Similarly, non-adherence to the lockdown rules was documented during the month of April 2020 and before lockdown was lifted.
- For variables measured in secondary mental health services, we tested hypotheses by assigning the pre-, during and post-lockdown cohorts by booking date rather than date of referral to secondary mental health services to further check the soundness of our construction of the models for analysis. We anticipated that there would be significant sensitivity to the date of any actual effect due to the rapid change in policy responses to the COVID-19 pandemic, so it was important to check the validity of our assumptions in constructing models for statistical analysis.

The results from the sensitivity analyses are reported below in the supplementary materials: tables 2.1 and 2.2.

*Results*

Sensitivity analyses showed little change in the significance of reported results when excluding the weeks before and after the start and lift of the UK COVID-19 lockdown period or altering the date of the start of lockdown, but the estimated effect sizes varied considerably (supplementary materials, tables 2.1). Variation in all the outcome variables measured in secondary mental health services due to the UK lockdown was affected using booking date to calculate the pre-, during and post-lockdown groups: if women’s inclusion in the during lockdown or post-lockdown group is calculated by their first antenatal appointment rather than the date they were referred to secondary mental health services, there is no evidence of an increase in the odds that women experienced virtual consultations with mental health services, and there was only a small drop in the odds that women were recorded to have experienced DVA during pregnancy in the during lockdown cohort, not the post-lockdown group. This is expected, as for example, a woman who attended her first antenatal appointment in January 2020 but was referred in April 2020 would be included in the during lockdown cohort in the main analysis, but in the pre-lockdown group in the sensitivity analysis. Her experience of pregnancy during her referral to secondary mental health services would be more appropriately categorised as during lockdown, and the findings of our sensitivity analysis highlights the importance of classifying women’s experiences appropriately. The results of this sensitivity analysis are listed in the supplementary materials, table 2.2, and further description of how women were included in the pre- during or post-lockdown cohorts can be found in the Supplementary Materials 1: Data extraction and timepoints in pregnancies and Supplementary Materials: Figure 1.

**Supplementary Material Table 1.1**: Subgroup analysis – Frequency table showing rates of women referred to secondary mental health services and rates of women who were Whooley Positive by ethnicity and IMD quintile

| Variable | Pre lockdown (01/10/2018-22/03/2020) | | | Lockdown (23/03/2020-10/05/2020) | | | Post lockdown (11/05/2020-29/08/2020 | | | Full cohort (01/10/2018-29/08/2020 | | | |
| --- | --- | --- | --- | --- | --- | --- | --- | --- | --- | --- | --- | --- | --- |
|  | Total | n | % | Total | n | % | Total | n | % | Total | n | % |  |
| Whooley Positive, by maternal ethnicity | 19812 | 1806 | 9.4% | 1684 | 179 | 10.6% | 3479 | 302 | 8.7% | 24975 | 2341 | 9.4% |  |
| Other | 1140 | 110 | 9.6% | 139 | 17 | 12.2% | 241 | 24 | 10.0% | 1520 | 151 | 9.9% |  |
| Asian | 1274 | 103 | 8.1% | 121 | 14 | 11.6% | 196 | 12 | 6.1% | 1591 | 129 | 8.1% |  |
| Black | 4014 | 451 | 11.2% | 316 | 43 | 13.6% | 613 | 65 | 10.6% | 4943 | 559 | 11.3% |  |
| White | 9745 | 781 | 8.0% | 814 | 82 | 10.1% | 1637 | 141 | 8.6% | 12196 | 1004 | 8.2% |  |
| Chinese | 383 | 22 | 5.7% | 41 | <10 | 0% | 78 | <10 | 0.0% | 502 | 28 | 5.6% |  |
| Mixed | 899 | 109 | 12.1% | 78 | <10 | 0% | 170 | 16 | 9.4% | 1147 | 131 | 11.4% |  |
| Unknown | 2357 | 230 | 9.8% | 175 | 17 | 9.7% | 544 | 38 | 7.0% | 3076 | 285 | 9.3% |  |
| Whooley Positive, by IMD quintile | 19567 | 1782 | 9.1% | 1661 | 176 | 10.6% | 3446 | 300 | 8.7% | 24674 | 2258 | 9.2% |  |
| 1 | 3786 | 371 | 9.8% | 316 | 35 | 11.1% | 675 | 73 | 10.8% | 4777 | 479 | 10.0% |  |
| 2 | 8111 | 769 | 9.5% | 716 | 74 | 10.3% | 1476 | 120 | 8.1% | 10303 | 963 | 9.3% |  |
| 3 | 4964 | 439 | 8.8% | 424 | 46 | 10.8% | 854 | 83 | 9.7% | 6242 | 568 | 9.1% |  |
| 4 | 1874 | 131 | 7.0% | 143 | 17 | 11.9% | 311 | 19 | 6.1% | 2328 | 167 | 7.2% |  |
| 5 | 832 | 72 | 8.7% | 60 | <10 | 0.0% | 130 | <10 | 0.0% | 1022 | 82 | 8.0% |  |
| Referral to secondary mental health services, by maternal ethnicity | 19812 | 810 | 4.1% | 1684 | 64 | 3.8% | 3479 | 117 | 3.4% | 24975 | 991 | 4.0% |  |
| Other | 1140 | 31 | 2.7% | 139 | <10 | 0.0% | 241 | <10 | 0.0% | 1520 | 44 | 2.9% |  |
| Asian | 1274 | 35 | 2.7% | 121 | <10 | 0.0% | 196 | <10 | 0.0% | 1591 | 41 | 2.6% |  |
| Black | 4014 | 202 | 5.0% | 316 | 14 | 4.4% | 613 | 30 | 4.9% | 4943 | 246 | 5.0% |  |
| White | 9745 | 392 | 4.0% | 814 | 33 | 4.1% | 1637 | 51 | 3.1% | 12196 | 476 | 3.9% |  |
| Chinese | 383 | <10 | 0.0% | 41 | <10 | 0.0% | 78 | <10 | 0.0% | 502 | 11 | 2.2% |  |
| Mixed | 899 | 47 | 5.2% | 78 | <10 | 0.0% | 170 | <10 | 0.0% | 1147 | 54 | 4.7% |  |
| Unknown | 2357 | 95 | 4.0% | 175 | <10 | 0.0% | 544 | 18 | 3.3% | 3076 | 119 | 3.9% |  |
| Referral to secondary mental health services, by IMD quintile | 19567 | 804 | 4.1% | 1661 | 63 | 3.8% | 3446 | 116 | 3.4% | 24674 | 983 | 4.0% |  |
| 1 | 3786 | 168 | 4.4% | 316 | 17 | 5.4% | 675 | 25 | 3.7% | 4777 | 210 | 4.4% |  |
| 2 | 8111 | 380 | 4.7% | 716 | 28 | 3.9% | 1476 | 55 | 3.7% | 10303 | 463 | 4.5% |  |
| 3 | 4964 | 179 | 3.6% | 424 | 10 | 2.4% | 854 | 24 | 2.8% | 6242 | 213 | 3.4% |  |
| 4 | 1874 | 54 | 2.9% | 143 | <10 | 0.0% | 311 | <10 | 0.0% | 2328 | 68 | 2.9% |  |
| 5 | 832 | 23 | 2.8% | 60 | <10 | 0.0% | 130 | <10 | 0.0% | 1022 | 29 | 2.8% |  |

**Supplementary Materials Table 1.2:** Subgroup Analysis - Estimated effects of the start of lockdown on 23/03/2020 and lift-of-lockdown announcements on 10/05/2020 for women who attended their first antenatal appointment between 1st January to 29th August 2020, estimating transitions related to the lockdown announcement (23rd March 2020) and lift-of-lockdown announcement (10th May 2020) on the rates of women who were Whooley positive and rates of referral to secondary mental health services by ethnicity and IMD. Odds ratios, correlation coefficients and 95% confidence intervals were estimated.

|  | Pre-lockdown vs. Lockdown | Pre-lockdown vs. Post-lockdown | Lockdown vs Post-lockdown |
| --- | --- | --- | --- |
|  | adjOR (95%CI) | adjOR (95%CI) | adjOR (95%CI) |
| *Whooley Positive, by maternal ethnicity* |  |  |  |
| Other (n=1094) | 1.41 (0.43-4.58) | 1.04 (0.40-2.72) | 0.74 (0.23-2.43) |
| Asian (n=1111) | 1.85 (054-6.34) | 0.78 (0.28-2.15) | 0.42 (0.12-1.44) |
| Black (n=3376) | 1.72 (0.95-3.14) | 0.83 (0.52-1.33) | 0.48 (0.27-0.88)* |
| White (n=8331) | 1.75 (1.12-2.71)* | 1.20 (0.84-1.72) | 0.69 (0.45-1.05) |
| Chinese | Not applicable, n is too low |  |  |
| Mixed (n=825) | 0.39 (0.107-1.40) | 0.47 (0.18-1.24) | 1.23 (0.31-4.27) |
| Unknown (n=2210) | 0.71 (0.31-1.66) | 0.60 (0.29-1.23) | 0.84 (0.38-1.86) |
| *Whooley Positive, by IMD quintile* |  |  |  |
| 1 (n=3311) | 1.23 (0.65-2.32) | 1.04 (0.622-2.32) | 0.51 (0.46-1.58) |
| 2 (n=7180) | 0.77 (0.54-1.10) | 0.78 (0.54-1.10) | 0.55 (0.36-0.86)** |
| 3 (n=4322) | 1.11 (0.62-1.97) | 0.99 (0.62-1.60) | 0.90 (0.51-1.56) |
| 4 (n=1598) | 7.82 (2.28-26.79)*** | 1.38 (0.55-3.46) | 0.18 (0.05-0.58)** |
| 5 (n=689) | 0.72 (0.14-3.59) | 0.92 (0.22-4.28) | 1.37 (0.28-6.68) |
| *Referral to secondary mental health services, by maternal ethnicity* |  |  |  |
| Other (n=973) | 2.87 (0.31-26.68) | 2.08 (0.35-12.54) | 0.72 (0.10-5.27) |
| Asian (n=977) | 4.94 (0.33-74.67 | 0.52 (0.06-4.54) | 0.10 (0.01-1.57) |
| Black (n=3376) | 1.22 (0.49-3.04) | 0.93 (0.46-1.89) | 0.77 (0.32-1.85) |
| White (n=8331) | 1.26 (0.67-2.34) | 0.88 (0.42-1.49) | 0.70 (0.38-1.29) |
| Chinese | Not applicable, n is too low |  |  |
| Mixed (n=739) | 0.29 (0.04-2.42) | 0.20 (0.04-0.95)* | 0.38 (0.09-5.21) |
| Unknown (n=2210) | 0.88 (0.23-3.45) | 0.58 (0.21-1.62) | 0.66 (0.18-2.39) |
| *Referral to secondary mental health services, by IMD quintile* |  |  |  |
| 1 (n=3311) | 1.08 (0.45-2.63) | 0.63 (0.30-1.34) | 0.58 (0.25-1.39) |
| 2 (n=7180) | 1.07 (0.55-2.07) | 0.72 (0.42-1.22) | 0.68 (0.36-1.26) |
| 3 (n=4322) | 0.81 (0.30-2.22) | 0.84 (0.39-1.81) | 1.03 (0.38-2.77) |
| 4 (n=1598) | 5.77 (0.84-39.57) | 1.24 (0.31-5.00) | 0.21 (0.03-1.38) |
| 5 (n=689) | 5.17 (0.31-85.38) | 5.98 (0.48-73.98) | 1.16 (0.12-11.07) |
| a Results from logistic regression models, with the three cohorts calculated from the date of the first antenatal appointment. Models are unadjusted due to low sample sizes. * p<0.05 ** p<0.01 *** p<0.001 | | | |

| **Supplementary Materials Table 2.1:** Sensitivity Analysis - Estimated effects of the start of lockdown and lift-of-lockdown announcements on women who attended their first antenatal appointment between 1st January to 29th August 2020, estimating transitions related to the lockdown announcement and lift-of-lockdown announcement. Odds ratios, correlation coefficients and 95% confidence intervals were estimated. | | | |
| --- | --- | --- | --- |
|  | **Pre-lockdown vs. Lockdown** | **Pre-lockdown vs. Post-lockdown** | **Lockdown vs Post-lockdown** |
|  | **Odds Ratio (95%CI)** | **Odds Ratio (95%CI)** | **Odds Ratio (95%CI)** |
| *Start of lockdown: 16^th^ March 2020* | | | |
| Whooley Positive ^a^ **n= 17292** | 1.30 (0.98-1.72) | 0.90 (0.71-1.15) | 0.70 (0.53-0.914)** |
| Help Required ^a^ **n= 1233** | 0.59 (0.31-1.16) | 0.99 (0.58-1.69) | 1.66 (0.88-3.14) |
| Antenatal DVA reported at booking ^a^ **n= 17292** | 1.83 (0.52-6.45) | 1.86 (0.61-5.71) | 1.02 (0.34-3.09) |
| Referral to secondary mental health services during pregnancy ^a^ **n= 17292** | 1.17 (0.76-1.80) | 0.79 (0.55-1.14) | 0.68 (0.45-1.02) |
| **Outcomes measured in secondary mental health services** | | | |
| Virtual contact with secondary mental health services during pregnancy (binary) ^b^**, n=657** | 1.82 (0.60-5.69) | 2.69 (1.22-5.92)* | 1.47 (0.57-4.12) |
| Face-to-face contact with secondary mental health services during pregnancy (binary) ^b^**, n=657** | 0.76 (0.24-2.42) | 1.04 (0.46-2.33) | 1.37 (0.40-2082) |
| DVA recorded in secondary mental health services during pregnancy ^b^**, n=657** | 0.16 (0.04-0.54)** | 0.43 (0.18-1.01) | 2.71 (0.92-8.02) |
|  | **Correlation Coefficient (95%CI)** | **Correlation Coefficient (95%CI)** | **Correlation Coefficient (95%CI)** |
| Frequency of f2f contact with secondary mental health services during pregnancy ^c^**, n=312** | -2.13 (-7.03-2.75) | 1.71 (-3.17-6.60) | 3.85 (-0.39-8.09) |
| Frequency of virtual contact with secondary mental health services during pregnancy ^c^**, n=657** | 3.40 (-1.31-8.11) | 1.25 (-3.37-5.87) | -2.15 (-3.12-1.83) |
| *Weeks beginning 16/03/20, 23/03/20, 04/05/20 and 11/05/20 omitted* | | | |
| Whooley Positive ^a^ **n= 17292** | 1.40 (1.05-1.87)* | 0.924(0.73-1.17) | 0.660(0.50-0.88)** |
| Help Required ^a^ **n= 1233** | 0.61 (0.31-1.21) | 1.01 (0.59-1.73) | 1.66 (0.86-3.24) |
| Antenatal DVA reported at booking ^a^ **n= 17292** | 1.36 (0.38-4.92) | 1.49 (0.52-4.30) | 1.09 (0.33-3.62) |
| Referral to secondary mental health services during pregnancy ^a^ **n= 17292** | 1.19 (0.77-1.86) | 0.780 (0.55-1.12) | 0.66 (0.43-1.00) |
| **Outcomes measured in secondary mental health services** | | | |
| Virtual contact with secondary mental health services during pregnancy (binary) ^b^**, n=657** | 1.82 (0.59-5.69) | 2.69 (1.22-5.93)* | 1.47 (0.53-4.12) |
| Face-to-face contact with secondary mental health services during pregnancy (binary)^b^**, n=657** | 0.76 (0.24-2.42) | 1.04 (0.46-2.33) | 1.37 (0.49-3.82) |
| DVA recorded in secondary mental health services during pregnancy ^b^**, n=657** | 0.16 (0.046-0.54)** | 0.43 (0.18-1.01) | 2.71 (0.92-8.02) |
|  | **Correlation Coefficient (95%CI)** | **Correlation Coefficient (95%CI)** | **Correlation Coefficient (95%CI)** |
| Frequency of f2f contact with secondary mental health services during pregnancy ^c^**, n=412** | 0.36 (-3.69-4.41) | 1.58 (-1.18-4.33) | 1.22 (-2.46-4.89) |
| Frequency of virtual contact with secondary mental health services during pregnancy ^c^**, n=300** | -0.01 (-0.405-4.02) | -0.67 (-3.43-2.09) | -0.66 (-4.35-3.04) |
| ^a^ Results from logistic regression models adjusted for maternal ethnicity, monthly trends and trends over different days of the week, with the three cohorts calculated from the date of the first antenatal appointment ^b^ Results from logistic regression models adjusted for monthly trends and trends over different days of the week, with the three cohorts calculated from the date of the first referral during pregnancy to secondary mental health services ^c^ Results from OLS regression models adjusted for monthly trends and trends over different days of the week with the three cohorts calculated from the date of first contact with secondary mental health services  * p<0.05 ** p<0.01 *** p<0.001 | | | |

| **Supplementary Materials Table 2.2:** Sensitivity Analysis - Estimated effects of the start of lockdown on 23^rd^ March 2020 and lift-of-lockdown on 10^th^ May 2020 announcements on women who attended their first antenatal appointment between 1st January to 29th August 2020, estimating transitions related to the lockdown announcement and lift-of-lockdown announcement by the date of the first antenatal appointment . Odds ratios, correlation coefficients and 95% confidence intervals were estimated. | | | |
| --- | --- | --- | --- |
|  | Pre-lockdown vs. Lockdown | Pre-lockdown vs. Post-lockdown | Lockdown vs Post-lockdown |
|  | Odds Ratio (95%CI) | Odds Ratio (95%CI) | Odds Ratio (95%CI) |
| Virtual contact with secondary mental health services during pregnancy (binary) ^b^**, n=657** | 0.35 (0.26-1.34) | 1.42 (0.36-3.07) | 2.18 (0.89-5.36) |
| Face-to-face contact with secondary mental health services during pregnancy ^b^**, n=657** | 0.62 (0.24-1.60) | 1.15 (0.54-2.43) | 1.86 (0.74-4.70) |
| DVA recorded in secondary mental health services during pregnancy (binary) ^b^**, n=657** | 0.34 (0.12-0.96)* | 0.56 (0.25-1.27) | 1.65 (0.61-4.46) |
|  | **Correlation Coefficient (95%CI)** | **Correlation Coefficient (95%CI)** | **Correlation Coefficient (95%CI)** |
| Frequency of f2f contact with secondary mental health services during pregnancy ^c^**, n=657** | 2.89 (-2.40-8.18) | -1.05 (-5.40-3.30) | -3.94 (-9.16-1.28) |
| Frequency of virtual contact with secondary mental health services during pregnancy ^c^**, n=361** | 4.42 (-0.39-9.24) | 2.04 (-1.77-5.84) | -0.66 (-4.35-3.04) |
| ^a^ Results from logistic regression models adjusted for maternal ethnicity, monthly trends and trends over different days of the week, with the three cohorts calculated from the date of the first antenatal appointment ^b^ Results from OLS regression models adjusted for monthly trends and trends over different days of the week with the three cohorts calculated from the date of first antenatal appointment.  * p<0.05 ** p<0.01 *** p<0.001 | | | |

**Supplementary Materials 4: Members of the eLIXIR Partnership**

**Professor Lucilla Poston,** Tommy's Professor of Maternal & Fetal Health, Department of Women and Children’s Health, School of Life Course Sciences, King’s College London.

**Professor David Edwards,** Chair in Paediatrics & Neonatal Medicine, Department of Perinatal Imaging and Health, King’s College London. Neonatal Consultant at Guy’s and St. Thomas’ NHS Foundation Trust.

**Professor Robert Stewart,** Professor of Psychiatric Epidemiology & Clinical Informatics, Department of Psychological Medicine, Institute of Psychiatry, Psychology and Neuroscience, King’s College London and NIHR Maudsley Biomedical Research Centre, South London and Maudsley NHS Foundation Trust, London. Consultant Psychiatrist at South London and Maudsley NHS Foundation Trust, London.

**Professor Louise M Howard,** Professor of Women’s Mental Health, Section of Women’s Mental Health, Institute of Psychiatry, Psychology and Neuroscience, King’s College London and NIHR Maudsley Biomedical Research Centre, South London and Maudsley NHS Foundation Trust, London. Consultant Psychiatrist at South London and Maudsley NHS Foundation Trust, London.

**Dr Mark Ashworth,** Reader of Primary Care, Department of Population Health Sciences, School of Population Health & Environmental Sciences, King’s College London.

**Professor Jane Sandall,** Professor of Social Science & Women’s Health, Department of Women and Children’s Health, School of Life Course Sciences, King’s College London.

**Professor Francesca Happé,** Professor of Cognitive Neuroscience, Social Genetic and Developmental Psychiatry Centre, Institute of Psychiatry, Psychology and Neuroscience, King’s College London.

**Professor Tim Spector,** Professor of Genetic Epidemiology, Department of Twin Research & Genetic Epidemiology, School of Life Course Sciences, King’s College London.

**Professor Andrew Shennan,** Professor of Obstetrics**,** Department of Women and Children’s Health, School of Life Course Sciences, King’s College London and Obstetric Consultant at Guy’s and St. Thomas’ NHS Foundation Trust.

**Professor Seeromaine Harding,** Professor of Social Epidemiology & Nutrition, Department of Diabetes, School of Life Course Sciences, King’s College London.

**Professor Anne Greenough,** Professor of Neonatology and Clinical Respiratory Physiology, Department of Women and Children’s Health, School of Life Course Sciences, King’s College London and Neonatal Consultant at King’s College Hospital NHS Foundation Trust.

**Dr Ingrid Wolfe,** Clinical Senior Lecturer, Department of Women and Children’s Health, School of Life Course Sciences, King’s College London and Consultant in Children's Public Health Medicine and Director of the Evelina London Children’s Healthcare.

**Dr Lauren Carson,** Postdoctoral Research Project Manager, Department of Psychological Medicine, Institute of Psychiatry, Psychology and Neuroscience, King’s College London.

**Ms Amanda Grey,** Lay member of the eLIXIR Oversight Committee.

**Dr Cheryl Gillett,** Head of Tissue Banking, Department of Comprehensive Cancer Centre, School of Cancer & Pharmaceutical Sciences, King’s College London.

**Ms Claire Delaney-Pope**, Head of Information Governance, South London and Maudsley NHS Foundation Trust, London.

**Dr Grenville Fox,** Neonatal Consultant at Guy’s and St. Thomas’ NHS Foundation Trust.

**Professor Laura Magee,** Professor of Women’s Health, Department of Women and Children’s Health, School of Life Course Sciences, King’s College London.

**Dr Nick Kametas**, Maternal Fetal Medicine and Obstetrics Consultant at King’s College Hospital NHS Foundation Trust.

**Ms Laura McFarlane,** Director of the LEAP Programme, National Children’s Bureau.

**Dr Melita Irving,** Clinical Genetic Consultant at Guy’s and St. Thomas’ NHS Foundation Trust.

**Dr Michael Absoud,** Paediatric Consultant at Evelina London Children’s Healthcare.

**Mr Paul Seed,** Senior Lecturer in Medical Statistics, Department of Women and Children’s Health, School of Life Course Sciences, King’s College London.

**Ms Sarah Spring,** Lay member of the eLIXIR Oversight Committee.

**Dr Sesh Sunkara,** Clinical Senior Lecturer, Department of Women and Children’s Health, School of Life Course Sciences, King’s College London and Reproductive Medicine and Gynaecology Consultant at Guy’s and St. Thomas’ NHS Foundation Trust.

**Dr Edward Barker,** Reader, Centre for Population Neuroscience and Precision Medicine, Institute of Psychiatry, Psychology and Neuroscience, King’s College London.

**Dr Theodore Dassios,** Senior Lecturer, Department of Women and Children’s Health, School of Life Course Sciences, King’s College London and Neonatal Consultant at King’s College Hospital NHS Foundation Trust.

**Dr Theresa Reyes,** Neonatal and Paediatric Consultant at King’s College Hospital NHS Foundation Trust.

**Ms Amelia Jewell**, Clinical Data Linkage Service Lead, NIHR Maudsley Biomedical Research Centre, South London and Maudsley NHS Foundation Trust.

**Mr Matthew Broadbent**, CRIS Clinical Informatics Lead, NIHR Maudsley Biomedical Research Centre, South London and Maudsley NHS Foundation Trust, London

**Dr Angela Flynn**, Postdoctoral Research Associate, Department of Women and Children's Health, School of Life Course Sciences, King's College London

1. Moscoe E, Bor J, Bärnighausen T. Regression discontinuity designs are underutilized in medicine, epidemiology, and public health: A review of current and best practice. J. Clin. Epidemiol. 2015; **68**: 132–43. [↑](#footnote-ref-1)
